# Acknowledgment to Reviewers of *Brain Sciences* in 2020

**DOI:** 10.3390/brainsci11020138

**Published:** 2021-01-22

**Authors:** Brain Sciences Editorial Office

**Affiliations:** MDPI AG, St. Alban-Anlage 66, 4052 Basel, Switzerland

Peer review is the driving force of journal development, and reviewers are gatekeepers who ensure that *Brain Sciences* maintains its standards for the high quality of its published papers. Thanks to the cooperation of our reviewers, in 2020, the median time to first decision was 17 days and the median time to publication was 35 days. The editors would like to express their sincere gratitude to the following reviewers for their precious time and dedication, regardless of whether the papers were finally published:
Aarabi, ArdalanLisowska, BarbaraAbate, GiuliaListos, JoannaAbbate, VincenzoLiu, HangfanAcerbi, FrancescoLiu, NingAcquaviva, EricLiu, SaAdams, Brian D.Liu, WeiAdams, Katie SmithwickLiu, XiuyunAddicott, MeridethLiu, YuanAdina, Turcu-StiolicaLiu, ZhiyongAfrantou, TheodoraLlano, DanielAfzal, Muhammad RaheelLlobet, ArturAgbangla, NounagnonLo Iacono, Cristina Anna MariaAghajani, HalehLodha, NehaAguillon-Hernandez, NadiaLoebel, FranziskaAhlström, ChristerLoewy, JoanneAhmad, FahimLohoff, FalkAhmad, KhurshidLoi, Samantha M.Ahmed, ZubairLojo-Seoane, CristinaAhn, HyocholLombardi, AngelaAikins, DeaneLong, ElizabethAkbar, MohammedLópez Puga, JorgeAkwa, YvetteLópez-Higes, RamónAl Zoubi, ObadaLópez-Martínez, Jesús AlbertoAlbouy, PhilippeLorenc, ElizabethAlexeyev, MikhailLotze, MartinAlfian, GanjarLourbopoulos, AthanasiosAlfieri, PaoloLoveall, SusanAl-Gharaibeh, AbeerLozano, ReymundoAl-Hilaly, YoussraLozeron, PierreAl-Janabi, ShahdLu, CalvinAllé, Mélissa C.Lu, ChiahaoAllen, PhilipLuca, FalzoneAl-Nuaimi, Ali H. HusseenLucchese, GuglielmoAlonzo, DanaLucchiari, ClaudioAlqahtani, AbdullahLugo, Ricardo GregorioAlquezar-Burillo, CarolinaLugo-Candelas, ClaudiaAlsharairi, NaserLui, FaustaAl-Shargie, FaresLuke, DavidAlwabil, AreejLundqvist, ChristoferAlyahya, MossaedLundstrom, Brian NilsAmaricai, ElenaLuo, ZonghuaAmoroso, NicolaLuongo, LivioAmu, SylvieLupo, GiuseppeAn, JinungLur, GyorgyAndereggen, LukasLuthar, SuniyaAndersen, Zorana JovanovicLuyat, MarionAnderson, JanisLuzzi, SabinoAndica, ChristinaLyberg Åhlander, VivekaAndjelkovic, Anuska V.Lynch, CharlesAndrade, PabloLysle, Donald T.Andreou, MariaLystad, Reidar P.Andreu-Sánchez, CeliaMa, DaAndriessen, KarlMa, LiangsuoAndrillon, ThomasMacey, PaulAngioni, Maria MaddalenaMaciejczyk, MateuszAnglada-Huguet, MartaMaciukiewicz, MalgorzataAnnen, JitkaMadureira, Patrícia A.Antohe, Magda-EcaterinaMaffei, ChiaraAntoine, PascalMahfouda, SimoneAnund, AnnaMakowski, NathanAoki, TomohiroMakuuchi, MichiruAppelbaum, Lawrence GregoryMalaguarnera, MicheleAquaro, StefanoMalinowska, UrszulaAragón-Vela, JerónimoManassi, MauroAravamuthan, Bhooma R.Manchaiah, VinayaArboix, AdriàManford, MarkArcara, GiorgioMånsson, SvenArdalan, MaryamManzano, RaquelArmstead, William M.Manzini, IvanArnau, StefanManzo, CiroArnulfo, GabrieleMaoz, UriAroni, SoniaMapelli, LisaAschenbrenner, Andrew J.Marano, MassimoAscoli Marchetti, AndreaMarbacher, SergeAseervatham, JayaMarcovitch, StuartAshour, HossamMarden, Franklin A.Assenza, GiovanniMarginǎ, DenisaAtwood, Brady K.Marino, SilviaAugustyniak, PiotrMarion, Marie-HeleneAurore, PerraultMarkowicz-Piasecka, MagdalenaAust, SabineMaroteaux, LucAvgerinos, KonstantinosMarotta, RosaAvignone, ElenaMarra, Alberto M.Axisa, Pierre-PaulMarshman, Laurence A. G.Azevedo, RubenMartelletti, PaoloBabcock, Julia C.Martín, JavierBączyk, MarcinMartin, Paula I.Baddam, SumanMartinez Ferreiro, SilviaBadesa, Francisco JavierMartínez-Banaclocha, Marcos ArturoBaez-Nieto, DavidMartinez-Horta, SaúlBaglioni, ValentinaMartinez-Pujalte, AntonioBagyinszky, EvaMartínez-Rodríguez, AliciaBaird, BenjaminMartin-Fernandez, MarioBaizabal-Carvallo, José FidelMartini, DouglasBakhshinejad, AliMartinkova, PatriciaBalaban, Daniel VasileMarzocchi, Gian MarcoBalboni, GiuliaMasamoto, KazutoBalestra, CostantinoMasero, ValentinBalestrini, SimonaMasland, SaraBalgiu, Beatrice AdrianaMassart, AlainBall, TonioMassey, ThomasBallard, KirrieMastroianni, AntonioBalota, DavidMastropietro, AlfonsoBaltuch, Gordon H.Mataric, Maja J.Balzano, TizianoMatowicka-Karna, JoannaBando, YoshioMatrone, CarmelaBanerjee, AnweshaMatsumura, TsuyoshiBansal, KanikaMaugeri, RosarioBao, ShanchengMaurer, LeonieBarbeau, EliseMaurus, IsabelBarceló, FranciscoMaye, AlexanderBarker, Felix M.Mayer, GeertBarker-Haliski, MelissaMazza, MonicaBarkley-Levenson, AmandaMazzone, LuigiBarløse, MadsMazzone, PaoloBarnes, Gregory N.McBride, Devin W.Barone, RitaMcCabe, Brian J.Baroni, StefanoMcCambridge, AlanaBarquín, Roberto RuizMcCarthy, MichaelBarquinero, JordiMcCrum, ChrisBarr, PeterMcDonough, Ian M.Barra, AdrianoMcFadden, SandraBarresi, GiacintoMcHugh, PatrickBarrett, DougMcKay, GarethBarrios-Fernández, SabinaMcKechanie, Andrew G.Bartfai, AnikoMcKenna, Peter EdwardBartoli, FrancescoMcNab, FionaBartsch, LeaMcPhillips, MartinBasavarajappa, Balapal S.Meabon, James S.Basirat, AnahitaMeehan, SeanBasnet, Ram ManoharMehaffey, HamishBatla, AmitMeier, JilBattaglino, Ricardo A.Meissner, AnjaBaumgartner, JosefMekary, SaidBaxter, BryanMelhem, Nadine M.Bayless, SarahMelie Garcia, LesterBazhanova, Elena D.Melkas, SusannaBazil, JasonMelogno, SergioBeasley, ShannonMeloni, BrunoBecchetti, AndreaMeltzoff, AndrewBecker, BenjaminMemo, MaurizioBecker, Jérôme A. J.Méndez-Álvarez, EstefaníaBégel, ValentinMendoza-Nuñez, Victor ManuelBeissner, FlorianMeneguzzo, PaoloBejarano-Martín, ÁlvaroMeng, YingBekkouch, Imad Eddine IbrahimMeng, YuanBekteshi, SarandaMengarelli, AlessandroBélanger, KasandraMerlino, GiovanniBell, RichardMesarwi, OmarBellato, AlessioMessenio, DarioBello-Espinosa, Luis E.Meszaros, MartinaBelmin, JoëlMewton, LouiseBenitez-Porres, JavierMeyer, AaronBenoit-Marand, MarianneMeyer, Heidi CatherineBerbrayer, DavidMeyners, MichaelBerke, BrettMiano, SilviaBerkers, RuudMichalopoulos, KostasBerman, Brian D.Mielcarek, MichalBernardi, GiulioMihaila, IuliaBernitsas, EvanthiaMikami, YoshinoriBernstein, Lori J.Miller, Christopher B.Bernucci, MarcelMiller, ElżbietaBertenthal, Bennett I.Miller, Judith S.Berthold, AnneMiller, KatherineBertrand, JacquelynMiller, Robert H.Bessi, ValentinaMillet, BrunoBest, RichardMills, Kelly A.Beutel, Manfred E.Milosavljevic, BosiljkaBhattacharyya, SaugatMin, KyunghaBhutta, Muhammad RaheelMinhas, Jatinder S.Bian, DayiMinic, ZeljkaBianchi, Vittorio EmanueleMiniero, Daniela ValeriaBianco, Maria GiovannaMirabella, GiovanniBiasini, Fred J.Misaki, MasayaBiegon, AnatMishra, DushyantBiehl, Stefanie C.Mishra, Jay S.Bieleninik, ŁucjaMishra, JyotiBienboire-Frosini, CecileMitra, JoyBies, Alexander J.Miyagishi, HirokoBijata, MonikaMiyamoto, YukiBikson, MaromMiyan, JaleelBin Azhar, M. A. HannanMiziak, BarbaraBinder, MarekMizuguchi, MasashiBingman, Verner PeterMizutani, KenmeiBini, FabianoMobbs, Ralph JasperBioulac, StephanieMoccia, MarcelloBirkett, MelissaModi, JigarBirney, DamianMohagheghi, AmirBisson, Marie-JoseeMohan, AdithBittker, Seth S.Mohsen, AttayebBlack, SheilaMolero-Jurado, María Del MarBlaine, SaraMolitch, MarkBlain-Moraes, StefanieMolteni, ErikaBlanchet, SophieMonaco, AlfonsoBland, Sondra T.Mondini, SaraBłaszczyszyn, MonikaMonroe, Derek C.Blesa, ManuelMonroy, ClaireBlinowska, Katarzyna J.Monteith, Teshamae S.Blume, FriederikeMontemurro, NicolaBoender, ArjenMontgomery, ErwinBogdan, MariaMontinari, Maria RosaBoling, Warren W.Montoya, EstrellaBologna, MatteoMoon, ChoozaBonaccorso, StefaniaMooney, SandraBonini, Sara AnnaMoont, RuthBoquete, LucianoMora-Jimenez, InmaculadaBorjini, NozhaMorand-Beaulieu, SimonBoronat-González, NuriaMoreland, Ashleigh T.Bortfeld, HeatherMoretti, AntimoBosman, Conrado A.Morioka, ShuBoström, KimMorita, TomoyoBotting, NicolaMorlet, ThierryBottini, GabriellaMorley, James F.Bottini, SilviaMorrier, Michael J.Botturi, AndreaMorris, KeithBoucart, MurielMorris, RosieBouhrara, MustaphaMorrow, LeslieBoulanger, MathieuMoscoso, AnaBousiges, OlivierMoscote-Salazar, Luis RafaelBovier, EmilyMosley, Philip E.Bower, James H.Mostert, Jeanette C.Bowling, Heather L.Mou, YongchaoBowyer, Susan M.Mounir, BendahmaneBoyd, Norman F.Mousavi Kahaki, Seyed MostafaBoyko, AlexeyMouzaki, AthanasiaBoys, ChristopherMuccio, RosyBozzato, PaoloMudigoudar, Basanagoud D.Bracco, MartinaMueller, Wolf C.Bradbury, AllisonMuffato, VeronicaBrahmajothi, Mulugu V.Mulak, AgataBrai, EmanueleMulherkar, ShalakaBrajša-Žganec, AndrejaMüller, Christian P.Bramham, CliveMüller, JulianeBramoweth, Adam D.Müller, ThomasBrandeis, DanielMullholland, PatrickBrasic, JamesMumaw, RandyBratitsis, TharrenosMurphy, RonanBraun, RalfMurray, JadeBrehm, AlexMurtaugh, MaureenBrenner, Colleen A.Musienko, PavelBrighina, FilippoMussey, Joanna L.Brogger, JanMutanen, Tuomas P.Bromberg, Mark B.Muthuraman, MuthuramanBrooks, Alyssa T.Muto, VincenzoBrouwer, Wiebo H.Mutoh, TatsushiBrown, Katlyn E.Mwilola, Patricia N.Brown, Matt J. N.Myers, Mark H.Brown, RussellN’Gouemo, ProsperBrüggemann, NorbertNagai, MutsumiBrühl, AnnetteNagasawa, ToshihikoBrumback, TyNaik, Ganesh R.Bruña, RicardoNair, SreenathBruno, JenniferNakagawa, TakayukiBruschini, LucaNakagomi, TakayukiBrustovetsky, NickolayNakajima, KenichiroBucci, Maria PiaNakamura, YukoBuckley, JeffreyNakasato, NobukazuBucova, MariaNakayama, HaruoBudimirovic, DejanNakisa, BaharehBugiotti, FrancescaNalivaiko, EugeneBuján, AnaNambu, IsaoBunketorp-Käll, LinaNanaware, PadmaBurchiel, Kim J.Narayana, ShaliniBurger, Michael C.Nariai, TadashiBurgess, Harold A.Narita, AkihiroBurghes, ArthurNarloch, JerzyBurgos-Robles, AnthonyNaro, AntoninoBurgunder, Jean-MarcNarzisi, AntonioBurke, RichardNashine, SonaliBurkhouse, Katie L.Naskar, ShovanBurnette, William BryanNassogne, Marie-CécileBury, SimonNastri, LiviaBusch, AlbertNat, Irina RoxanaBuskila, YossiNataf, SergeButler, TracyNatale, GianfrancoButti, NiccolòNatarajan, SivaramanByl, NancyNath, AudreyCabrera, Juan LuisNayak, ArnabCacciola, AlbertoNazir, Tatjana A.Cacialli, PietroNegro, AndreaCaetano, Gina MariaNegwer, ChiaraCaffò, AlessandroNekludov, MichaelCahill, MichaelNelson, AimeeCain, MatthewNemzer, Louis R.Calvo, AndreaNenov, MiroslavCampagnoli, RafaelaNetoff, TayCampagnolo, MartaNeueder, AndreasCampana, GianlucaNeulen, AxelCampbell, DanielNevšímalová, SoňaCampbell, Susan L.Newell, KarlCampesi, IlariaNewman, ChristopherCampi, CristinaNg, Kheng SiangCamponogara, IvanNg, Qin XiangCañas Delgado, José JuanNguyen, Van PhucCancer, AliceNiazi, Imran KhanCanessa, NicolaNicita, FrancescoCannon, JoannaNicoladis, ElenaCano-Espinosa, CarlosNicolas, NicastroCantone, MariagiovannaNicolletti, FerdinandoCaparros-Gonzalez, Rafael A.Niendam, TaraCapek, StepanNieto-Escamez, Francisco A.Capirci, OlgaNiimi, MasachikaCappellini, GermanaNikander, Riku W.Caprì, TindaraNikolaidis, AkiCaputo, CarmelaNikolin, StevanCardeña, EtzelNimsky, ChristopherCardinali, LucillaNing, LipengCariello, Annahir N.Niort, JannickCarmela, Di LucianoNishimura, KojiCarnevale, LorenzoNitta, AtsumiCaron, GuillaumeNizhnikov, Michael E.Carrano, AnnaNoé, EnriqueCarrus, ElisaNomura, TaishinCarson, RichardNopoulos, Peggy C.Carter, BenjaminNotaras, Michael J.Carver, Chase M.Novikova, IrinaCass, JohnNowaczewska, MagdalenaCastaneda Vega, SalvadorNowak, Michal S.Castillo, Carlos A.Nowakowska, AnnaCastillo, FranciscoNowicki, GrzegorzCastro, Maria AntónioNozohouri, SaeidehCatalano, PeterNugent, KennethCaudle, MichaelNuñez, AngelCaziuc, AlexandraNyquist, PaulCentanni, Tracy M.O’Donnell, Brian F.Cepeda, SantiagoO’Donnell, LaurenCeranowicz, PiotrO’Keeffe-Ahern, JonathanCerino, StefaniaO’Shaughnessy, DouglasCervera, Ignacio Campoy Oakes, Lisa M.Ceyte, HadrienObaid, MajedChakraborty, DarpanObuchi, ChieChalah, Moussa AntoineOchi, ShinichiroChamine, IrinaOgonowska, AnnaChandler, Melanie J.Ohbuchi, ToyoakiChang, Chi-YuanOhnishi, MasatoshiChang, KoyinOhno, JunChapman, KennethOhtsuki, GenCharalambous, CharalambosOkada, YoheiCharest, JonathanOkamoto, CurtisCharles, DavidOkamura, HisayoshiCharvet, LeighOlbrich, EckehardChatterjee, AnjanOlivares-Olivares, PabloChatzaki, EkateriniOlmos, Joan GuardiaChaudhary, UjwalOlsen, Michelle L.Chauhan, MunishOmlin, XimenaChekenya, MarthaOng, Jia HoongChen, ChongOrion, DavidChen, Feng-TzuOrlandi, AndreaChen, GuifenOrnello, RaffaeleChen, HansenOrtells, Juan JoseChen, JingOrtiz, J. BryceChen, Kao-ChinOrtolano, SaidaChen, Kuan-FuOsooli, MehdiChen, LiminOssmy, OriChen, ZhenOstrowski, RobertChenane, KathiaOsuagwu, BethelCheng, Chen-chuOvsepian, Saak V.Cheng, JiaOwens, Gregory P.Cheron, GuyOzbay, PinarChertkow, HowardOzburn, AngelaChesney, EdwardPaap, Kenneth R.Chester, Julia A.Padilla, MarielaChiarotti, FlaviaPagamenti, PaoloChiva-Bartoll, ÓscarPagé, GabrielleCho, Yu-MinPagel, James F.Choate, Peter W.Pal, AjayChoi, Doo-SupPalazón-Bru, AntonioChoi, HojongPalmiero, MassimilianoChoisdealbha, Áine NíPalmour, RobertaChoo, Pei LingPalumbo, RoccoChristian, CatherinePan, Ming-KaiChristiner, MarkusPanagis, GoergeChristou, EvangelosPane, MarikaChristova, PekaPang, Cheng-YoongChrondogianni, VickyPangrazzi, LucaChroust, Alyson J.Pankratov, YuriyChua, Koon Jiew EriPao, Ping-ChiehChung, Tae NyoungPaolicelli, DamianoChung, Yong-AnPapadimas, George KonstantinosChurch, Frank C.Papale, Andrew E.Chye, YannPapanagiotou, PanagiotisCiarambino, TizianaParacchini, SilviaCiaramitaro, VivianParameswaran, JananiCieslik, MagdalenaParanjape, AnuragCilene, RodriguesParikh, Pranav J.Cinel, CaterinaParis, HunterClarkson, BenjaminPark, DonghwiClifton, CharlesPark, JoongkyuCoenen, MaraikeParker, JackCoffman, Brian A.Parnetti, LucillaCohen, KobyParsons, TravisCohen-Salmon, MartinePartanen, Juhani V. S.Colic, LejlaPasquini, MassimoColon Saez, JosePassmann, SvenColón-Ortiz, Abner J.Passos, MarisaColunga, ElianaPastor, JesúsComi, CristoforoPaszkiel, SzczepanConci, MarkusPatel, BhavanaCondro, IgnatiusPaterson, KevinConinx, KarinPathak, DhrubaConner, JerushaPathak, Salil S.Constantinides, Vasilios C.Pati, DipaContet, CandicePatowary, AshokConti, AlanaPatten, Scott B.Conti, DanielaPatterson, John D. Conversi, DavidPatterson, Kristina R.Cooke, Bradley M.Paucar, MartinCooper, JeffreyPaudel, PradeepCooper, RobinPaun, Maria-AlexandraCooper-Knock, JohnathanPaun, Viorel-PuiuCopersino, MarcPavlova, NatalyaCoq, Jacques-OlivierPawlowska, BeataCorbetta, DanielaPayne, Jessica D.Corchs, SilviaPearlstein, TeriCordaro, MarikaPeelen, Marius V.Corrado, BrunoPeiffer-Smadja, NathanCorrado, GiovanniPeiris, CaseyCorrales-Serrano, MarioPelicioni, Paulo Henrique SilvaCorrigan, FrancesPendyala, Gurudutt N.Cortese, RosaPennicooke, BrentonCortesi, Paolo A.Pennington, CatherineCosentino, GiuseppePenzol, María JoséCosta, Anna MariaPepe, JessicaCostagli, MauroPercesepe, AntonioCouch, YvonnePerea, ManuelCoughlan, ChristinaPerera, Ricardo AugustoCoull, Bruce M.Peretti, SaraCoutellier, LaurencePerez Fernandez, Francisco Javier Craig, Sonya E. L.Pérez, David LópezCrawford, FionaPérez-Moreno, Juan JoséCressman, John R.Periasamy, RameshCriaud, MarionPerin, PaoloCrocamo, CristinaPeristeri, EleniCroce, PierpaoloPernet, CyrilCruice, MadelinePerri, Rinaldo LivioCsepany, TundePerrone, SerafinaCuenca-Martínez, FerranPerry, James R.Cummine, JacquelinePerry, Sarah E.Curcio, GiuseppePervin, MoniraĆurko-Cofek, BoženaPesce, AlessandroCurry, GwenettaPeschillo, SimoneCurtis, James A.Pesimena, GabrieleCury, ClairePeter-Derex, LaureCushing, Sharon L.Peterson, Steven M.Cutler, RoyPetit, LaurentCvetkovska, EmilijaPetrinovic, Marija-MagdalenaCzachowski, CristinePetrov, Megan E.Czosnyka, MarekPeyrin, CaroleD’Alonzo, MarcoPfeiffer, BethD’Amico, EmanuelePhung, Janice N.D’Arrigo, SoniaPiccione, MariaD’Atri, AuroraPickart, LorenD’Azzo, AlessandraPierce, Lisa M.D’Souza, DeanPiitulainen, HarriDa Silva Candal, AndrésPilloni, GiuseppinaDa Silva E Silva, DanielPina, MelanieDahm, Stephan F.Pincherle, AlessandroDai, YulinPineda, Jose RamonDaikoku, TatsuyaPini, LorenzoDaini, RobertaPini, Luigi AlbertoDakin, ChrisPinna, GrazianoDammers, RubenPinto, EdgarDannon, PinhasPiorecky, MarekDarby, KevinPiqueras, LauraDario, BrunettiPires De Lima, RafaelDas, UndurtiPiscitelli, DanieleDaskalakis, NikolaosPitts, TeresaDatta, Anita N.Placzek, MarysiaDatta, DibyadyutiPlatt, Donna M.Datta, HiaPlechawska-Wójcik, MałgorzataDavies, AlexanderPles, HoriaDavis, Mellar P.Plescia, FulvioDavis, Randall L.Plewan, ThorstenDavis, ThomasPoarch, Gregory J.De Aguiar, VâniaPodnar, SimonDe Beaurepaire, RenaudPoerio, GiuliaDe Bellard, Maria ElenaPoirel, NicolasDe Berardis, DomenicoPojo, MartaDe Carvalho, MamedePolańczyk, AndrzejDe Ceglie, VincenzoPolczynska, Monika M.De La Rosa, Antonio LuquePollok, BettinaDe La Torre, Gabriel G.Pölzl, GerhardDe Ridder, DirkPonce, ShainaDe Vries, JoostPons Rivero, Antonio JavierDeBuc, Delia CabreraPontillo, MariaDecavel, PierrePooresmaeili, ArezooDeclerck, MathieuPopa-Wagner, AurelDeeb, WissamPopugaeva, ElenaDelanoue, RenaldPorcaro, CamilloDelgado, Montserrat G.Porokhovnik, LevDella Vedova, Anna MariaPosar, AnnioDelpech, Jean ChristophePoston, BrachDelplanque, SylvainPoulin-Charronnat, BenedicteDemartini, LauraPountney, DeanDengler-Crish, ChristinePoznanski, PiotrDePoy, Lauren M.Prabhu, Sujit S.Descartes, MariaPradeepkiran, Jangampalli AdiDeshpande, LaxmikantPrakapenka, AlesiaDesideri, AlessandroPrashad, ShikhaDeth, Richard C.Prell, TinoDeuschl, GünterPrentice, HowardDevred, FrançoisPreston-Campbell, RebeccaDewey, Rebecca SusanPrete, GiuliaDhaher, RoniPreziosa, PaoloDhamala, MukeshPrieto, Anne L.Dhanasekaran, MuraliPrior, AnatDi Blasi, Francesco DomenicoProbst, Janice C.Di Flumeri, GianlucaProdi, NicolaDi Giovanni, PamelaProffitt, RachelDi Lazzaro, VincenzoProkop, Jeremy W.Diab, RamiProvini, FedericaDias, CelestePrunty, JonathanDiaz, MarvinPšemeneckienė, GretaDidangelos, TriantafyllosPuglisi, GuglielmoDiepers, MichaelPullen, ErinDifrancesco, MarkPushkarskaya, HelenDileonardi, Ann MaePutilov, ArcadyDing, NingPytel, PeterDing, RuiyingQian, MingxingDinh, Dzung H.Qin, LuyeDirin, AmirQin, PengminDiserens, KarinQuarantelli, MarioDissel, StephaneQuattrocchi, Carlo CosimoDoddapattar, PrakashQuattrone, AndreaDoddasomayajula, RaviQuílez-Robres, AlbertoDohrn, MaikeQuintana, David García Doi, HirokazuRabiger, JuttaDokos, SocratesRacz, AttilaDomagalska-Szopa, MalgorzataRad, Nastaran MohammadianDombrowski, YvonneRadakovic, RatkoDomenicali, MarcoRadke, AnnaDonaldson, NickRadowsky, Jason S.Donaldson, S. TiffanyRaffa, GiovanniDonatelli, GraziellaRafiad, IslamDonato, CarmelaRagazzoni, AldoDonato, LuigiRaggi, AlbertoDong, FengpingRahman, ShafiqurDong, QinglinRaikes, Adam C.Donskoy, InnessaRaimo, SimonaDoucet, Gaelle E.Rainero, InnocenzoDouris, PeterRalvenius, WilliamDowns, Jenny A.Ramdhani, RiteshDragomir, AndreiRamieri, GuglielmoDricu, MihaiRamirez, Paul MichaelDrigas, Athanasios S.Ramirez-Baena, LuciaDu, XiaomingRamírez-Moreno, José MaríaDuarte, AnaRamos, AdrianaDuce, JamesRamos, CarmenDuch, WłodzisławRango, MarioDuchemin, Anne-MarieRao, Sneha B.Dudok, JeroenRasero, JavierDuehlmeyer, LeonieRashid, UsmanDuker, Leah SteinRask-Andersen, HelgeDuncan, JohnRasova, KamilaDurant, SzonyaRaud, LiisaDussor, GregRavanidis, StylianosDyke, KatherineRavenscroft, JohnDymarek, RobertRavignani, AndreaDynia, JaclynRavona-Springer, RamitDyrba, MartinRazzaque, MohammedDzierżanowski, TomaszReader, ArranDziewas, RainerReddy, Sudhir PuttyEarhart, Gammon M.Reed, Tanea T.Eastman, Roger D.Reed, William R.Eaton, Misty J.Reeder, ReshanneEberhardt, OlafReedman, SarahEdelman, BradleyReeves, AdamEdgar, JuliaReichgelt, HanEdwards, Adam B.Reigal, RafaelEek, Meta N.Reiter, Lawrence T.Eggleston, JeffreyRener-Sitar, KsenijaEhler, JohannesRennels, Jennifer L.Ehrlich, DebraRespondek, GesineEhrlich, Stefan K.Revesz, DoraEichhorn, LarsRevuelta, Gonzalo J.Eigsti, Inge-MarieReynaud, EveEilam, DavidReynolds, Katharine C.Eilander, Henk J.Rhee, JoohyunEisenberg, Solomon R.Ricci, LorenzoEisenlohr-Moul, ToryRicciardi, LucianaEl Tahry, RiëmRich, Tonya L.Elia, MaurizioRichards, ToddEliseyev, AndreyRichardson, Heather N.El-Refaie, AmrRichman, DavidEmrani, SheinaRikkert, Marcel OldeEndres, DominikRiley, ZacharyEngemann, DenisRinker, John R.Engström, MariaRíos-Garcés, RobertoErnst, DanielRisher, Mary-LouiseErwin, William MarkRiva, ValentinaEscoffre, Jean-MichelRoberts, MarkEscolano-Pérez, ElenaRoberts, Rosalinda C.Esparham, AnnaRoberts, Todd F.Espín, LauraRobertson, Anne M.Esposito, GianlucaRobertson, FaithEsser, Michael J.Robinson, Stacey L.Evans, CarysRobson, Matthew JamesEvi, VerbecqueRocamora-Perez, PatriciaFabbri, ChiaraRoccella, MicheleFabbri-Destro, MaddalenaRocchi, LorenzoFabio, Rosa AngelaRočka, SauliusFacchin, AlessioRodger, AlisonFachim, Helene A.Rodrigues, AlexandreFaedda, NoemiRodrigues, Filipe B.Fagioli, FrancaRodrigues, JoãoFalkenstein, MichaelRodriguez, JavierFam, JustineRodríguez-Contreras, AdriánFang, QiRodriguez-Cuadrado, SaraFang, XingRogalska, JustynaFaria, Diego R.Rogatzki, MatthewFarnell, Damian J. J.Rogobete, Alexandru FlorinFatima, NidaRohleder, CathrinFattore, LianaRoldán, Ana María GonzálezFaull, OliviaRomano, CorradoFawcett, Angela J.Romero-Ayuso, DulceFehlings, Michael G.Romero-Collado, AngelFeldman, GaryRon-Angevin, RicardoFenesi, BarbaraRoncero, CarlosFeng, Hua-JunRosas, Herminia D.Ferguson, BradleyRosemann, StephanieFernández-Alcántara, ManuelRosenow, TimFernández-Castillo, AntonioRoso, Margarita CorominasFernandez-Vargas, JacoboRossi, SimoneFerrara, Nicole C.Rossi, SonjaFerree, Andrew W.Roth, RoswithFerreira, NelsonRoubertoux, PierreFerri, Sarah L.Rousset, FrancisFestini, SaraRovero, PauloFibert, PhilippaRowland, KimberlyFiehler, KatjaRoy, BhaskarField, MichaelRoy, Holly A.Filmer, Hannah L.Roy, Raphaëlle N.Fink, MaxRoyeen, CharlotteFinkel, DeborahRoza, Válber César CavalcantiFinsterer, JosefRozenkrantz, LironFiore, MarcoRozerta, SokouFioretti, AlessandraRubega, MariaFisher, Joseph A.Rubiano, Andres M.Fisher, Robert S.Rubiera, MartaFitch, Roslyn H.Ruda-Kucerova, JanaFlasbeck, VeraRudroff, ThorstenFleming, MelanieRuiz-Mezcua, BelénFogel, StuartRumney, PeterFogler, Kethera A.Ruotolo, FrancescoFontana, Andreia Cristina KarklinRussell, MatthewFornaro, MicheleRusted, JenniferForouzanfar, MohamadRyu, DuchwanForte, GiuseppeRzońca, Sylwia OlimpiaFouda, AbdelrahmanSaavedra, SandraFox, AdamSaba, LauraFranceschini, SandroSabisz, AgnieszkaFrank, SebastianSacheli, Lucia MariaFranzese, AdrianaSacrey, Lori-Ann R.Fraschini, MatteoSadegh-Zadeh, Seyed-AliFresnoza, ShaneSaelices, LorenaFreund, NadjaSafaei, SoroushFriedenberg, DavidSaft, CarstenFriedenberg, JaySah, NirnathFriedman, Joseph HaroldSahai, MichelleFriesdorf, RebeccaSaif, NabeelFrisina, RobertSaint-Pol, JulienFrolov, Roman V.Säisänen, LauraFroriep, Ulrich P.Saito, MarikoFu, XiaoxueSakakibara, HiroyukiFucà, RominaSakakibara, RyujiFuchs, ClaudiaSalari, SoniaFucile, SergioSalazar, AddissonFuerst, John G. R.Salman, MootazFujimoto, AyatakaSalmina, AllaFujioka, KazumichiSalvador, AliciaFujishima, IchiroSamadi, Sayyed AliFujita, KaoriSamadiani, NajmehFukuoka, TatsuyukiSampedro, PatriciaFulceri, FrancescaSamuel, Preethy S.Furse, SamuelSamuels, Bridget D.Fusar-Poli, LauraSanchez-Bornot, Jose MiguelGabriel, DamienSánchez-Campusano, RaudelGadad, Bharathi ShrikanthSanchez-Martinez, MelchorGaidin, Sergei G.Sánchez-Quinteiro, PabloGajavelli, ShyamSanchez-Varo, RaquelGalazyuk, AlexanderSanders, RossGaldi, PaolaSandoo, AamerGaleazzi, Gian MariaSanguinetti, Joseph L.Gałecki, PiotrSani, SepehrGalkin, Alexander S.Sankarasubramanian, VishwanathGalli, ManuelaSanna, FabrizioGallitto, GiuseppeSannar, Elise M.Gambaruto, AlbertoSanti, MariaritaGanchev, TodorSantos, LuisGandola, MartinaSaraiva, LindaGanis, GiorgioSarecka-Hujar, BeataGaravan, HughSaretzki, GabrieleGarbusow, MariaSargin, DeryaGarcía, Mario OrtizSarker, MarjanaGarcia, VictorSarkheil, PegahGarcía-Azorín, DavidSarubbo, Maria FiorellaGarcía-Díaz Barriga, GerardoSase, AjinkyaGarcía-Garnica, MarinaSasmita, Andrew OctavianGarcía-Iglesias, Juan JesúsSathyanesan, AaronGarcía-Massó, XavierSato, DaisukeGardella, ElenaSauter, MarianGarg, SukantSavostyanov, Alexander N.Garner, AngieSawicki, MarcinGarnham, AlanScalfari, AntonioGarofalo, StefanoScandola, MicheleGarrett, Mario D.Scarpelli, SerenaGarrote-Adrados, José AntonioSchade, HannahGascho, DominicSchallner, NilsGaum, Petra MariaScheerer, NicholeGaveau, JeremieSchellino, RobertaGavvala, JayScheres, Anouk. P. J.Gazerani, ParisaScherrer, Jeffrey F.Geminiani, AliceScheuer, TillGent, Thomas C.Schiavone, StefaniaGentile, EleonoraSchifano, FabrizioGeorge, NathalieSchiffer, FredricGeorgian, BadicuSchindler, LenaGeraci, FabianaSchindler-Ivens, SheilaGeraci, JosephSchipmann, StephanieGerbino, ValeriaSchlachetzki, JohannesGhag, GauravSchmitz, FlorianGhazi-Saidi, LadanSchmitz, JudithGhita, MihaelaSchneider, PeterGiamberardino, Maria AdeleScholkmann, FelixGiannakopoulos, PanteleimonSchramm, SeverinGibson, RaechelleSchriever, ValentinGigliotta, OnofrioSchroeter, MichaelGilbert, LeonaSchultz, StephenGiles, WayneSchupp, HaraldGill, ChandlerSchuss, PatrickGill, SimoneSchwarting, RainerGilmore, ColinSchwiedrzik, Caspar M.Giordan, EnricoScialò, CarloGiordano, CarlaSciaraffa, NicolinaGisquet-Verrier, PascaleSclocco, RobertaGiustetto, MaurizioScott, CobbGlenn, SheilaScott, Julia A.Glover, ElizabethScott, Rodney C.Glushakov, AlexanderSeasholtz, AudreyGobec, MartinaSebastiano, Davide RossiGobert, DeniseSeder, David B.Goedee, H. StephanSeeman, Mary V.Goehring, TobiasSefton, JoEllenGoel, RahulSegaert, KatrienGoffman, LisaSeichepine, DanielGoizet, CyrilSelleri, SilviaGolan, DanielSellers, KristinGold, WendySelvi, YavuzGolla, HeidrunSena, ArmandoGolomb, BeatriceSeo, Young JoonGomez, Christopher M.Serafini, SandraGomiero, TizianoSergeev, DmitryGonçalves, Ana RibeiroSergi, Pier NicolaGonzález, Julián J.Serrano, MercedesGonzalez-Castro, PalomaSeto, ToshiyukiGonzalez-Escamilla, GabrielSewaybricker, Leticia E.Goodday, SarahShafai, FakhriGoodman, MikeShah, PunitGopinath, KaundinyaShahani, UmaGorur-shandilya, SrinivasShahedi, MaysamGoudman, LisaShahin, AntoineGoudriaan, MarijeShan, HongmingGovindarajan, Sindhuja TirumalaiShan, Zack Y.Goyal, MayankSharma, NutanGoyal, RamanSharma, PrashantGracia-García, PatriciaSharma, RishiGraco, MarnieSharma, VerinderGradinaru, Andrei CristianSharma, Vibhash D.Gragnaniello, CristianSharon, DeniseGraña, ManuelShaw, LisaGranieri, EnricoSheets, Patrick L.Grant, RossSheikhbahaei, ShahriarGraves, JanessaSheldon, StevenGray, KatieShell, Courtney E.Greco, TiffanyShelley-Tremblay, JohnGrewal, Raji PaulShen, GuofangGriffin, WilliamShen, SanbingGrigoriev, AndreiShenolikar, ShirishGrimm, Marcus O. W.Shi, JieGrimm, OliverShi, YangmingGriñán-Ferré, ChristianShibata, YasushiGrippo, AntonelloShields, Grant S.Grochowski, CezaryShiina, AkihiroGroiss, StefanShim, Joon W.Grossbach, AndrewShimizu, KazuhideGrossi, EnzoShimizu, YukiGrube, SusanneShimodozono, MegumiGrubić Kezele, TanjaShin, Hae-WonGrundy, LukeShin, SuGruter, BasilShindo, AkihiroGu, BinShirakawa, TetsuoGu, FengShirin, SoniaGuadagni, VeronicaShirvalkar, PrasadGuediche, SaraShuffrey, Lauren C.Guerini, Franca R.Siddiquee, MasudurGuerraz, MichelSidiropoulos, ChristosGuha, SharmisthaSidtis, John J.Guillon, QuentinSigurdardottir, Heida MariaGuirado, RamonSigurdsson, HilmarGuirguis, AmiraSikka, PilleriinGuivier-Curien, CarineSilva Ribeiro, JoanaGulbis, JacquiSilva, LuísGulisano, MariangelaSilva, SusanaGüntürkün, OnurSimard, MarcGupta, VikasSimcock, GabrielleGurden, HiracSimon, Jessica R.Guzman, RaphaelSimos, PanagiotisHaar, ShlomiSimpson, ErickaHaarbauer-Krupa, JulietSimula, SakariHaas-Givler, BarbaraSin, AnthonyHacker, Mallory L.Singewald, NicolasHafez, AhmadSingh, AmardeepHahn, ChipSingh, ArunHahnloser, Richard H. R.Singh, ChanpreetHaider, HaulaSinha Ray, ShresthaHaider, NomanSiracusa, RosalbaHajra, Sujoy GhoshSirohi, SunilHall, Frank ScottSisoeva, OlgaHall, Toby M.Slaker, MeganHamann, MartineSleet, DavidHampson, David R.Sleigh, JamesHanas, JaySliwinska, AgnieszkaHanc, TomaszSłomka, ArturHanchate, NareshSmet, IrisHanna-Pladdy, BrendaSmirni, DanielaHannonen, RiittaSmith, Christopher J.Hansen, NielsSmith, DeanHansson, CarolineSmith, Glenn E.Hanstock, TanyaSmith, Heather F.Hanyuda, AkikoSmith, PaulHao, Hannah (Yu)Smith-Hicks, ConstanceHaro, GonzaloSnijders, TinekeHarper, JeremySobecki, JanuszHart, Ronald P.Socała, KatarzynaHartanto, AndreeSoderlund, GoranHärtl, RogerSoekadar, Surjo R.Hartley, CalumSohr-Preston, SaraHartman, Maria C.Sokhadze, EstateHartwig, JosephineSollmann, NicoHashmi, JaveriaSoltanifar, MohsenHassell, James E., Jr.Sommer, ClaudiaHattingen, ElkeSon, JongsangHattori, TsuyoshiSong, ChanghoHauser, Sheketha R.Song, LiujiangHawken, Emily R.Song, YeonsuHawn, Sage E.Song, YuyuHayashi, Mariko KatoSorensen, CarlHayden, Melvin R. (Pete)Soria-Frisch, AureliHayes, WayneSotgiu, StefanoHe, YongtianSpalice, AlbertoHecker, MichaelSpampinato, Danny A.Heckmann, JosefSpanoudis, GeorgeHector, Ralph D.Sparkenbaugh, Erica M.Heilbronner, UrsSpataro, RossellaHeller, ElizabethSpecchia, AntonioHellewell, SarahSpinu, LauraHemley, Sarah J.Spironelli, ChiaraHemmy, LauraSpreat, ScottHenderson, Michael X.Squillaro, TizianaHendrix, PhilippSrivastava, PranayHenneghan, Ashley M.Srivastava, SaurabhHepsomali, PirilSrivatsan, MalathiHerman, Pawel AndrzejStanciu, Gabriela-DumitritaHernan, AmandaStanfield, AndrewHerold, FabianStanziano, MarioHéroux, PaulStapleton-Kotloski, Jennifer R.Herrera, Alexandra YcazaStapor, KatarzynaHerrero, María JesúsStarke, Robert M.Hershey, LindaStasiolek, MariuszHertrich, IngoStavinoha, Peter L.Herzog, DavidStavropoulos, Katherine K. M.Hess-Dunning, AllisonStavrou, VasileiosHessels, RoySteardo, LucaHessl, DavidSteib, SimonHeyn, PatriciaSteiger, RuthHidalgo-Muñoz, Antonio R.Stein, JohnHigashi, HiroshiSteinberg, FabianHikmat, OmarStephan, FranziskaHilgenkamp, ThessaStephens, JaclynHilton, ClaudiaSternin, AvitalHinaut, XavierStessman, Holly A.Hindocha, ChandniStevens, Sandra L.Hingorani, DinaStewart, TessandraHinrichs, HermannStock, Ann-KathrinHinzen, WolframStocker, RetoHiraoka, KoichiStonerock, Gregory L.Hirsch, Mark A.Stoyanov, DrozdstojHo, MengfeiŠtrac, Dubravka ŠvobHo, RogerStrigaro, GionataHo, WinsonStrzalkowski, NickHocker, James R. S.Studenka, BreannaHocking, Darren R.Stuit, SjoerdHodgson, TimStutz, BernardoHodson, DanielleStylli, Stanley S.Hoerr, RobertSuckling, JohnHof, At L.Sudre, CaroleHoffart, AsleSuhr, FrankHoffman, PaulaSullivan, Aideen M.Hogenson, George B.Sullivan, Genevieve MaryHöller, YvonneSumien, NathalieHöllig, AnkeSummers, RebekahHoltzer, RoeeSun, NaweiHomman-Ludiye, JihaneSundaram, PadmavathiHondebrink, LauraSundaram, Sivaraj MohanaHori, HikaruSuñer, Damian HeineHori, SatoshiSung, Wen-WeiHorn, SebastianSuntrup-Krueger, SonjaHorton, CarolineSupek, SelmaHosford, Patrick S.Surges, RainerHoshi, AkihikoSuter, BernhardHossner, ErnstSutherland, MatthewHough, LyonSutton, JillHowell, OwainSwanson, Meghan R.Howlin, PatriciaŚwitońska, MilenaHramov, Alexander E.Szczepankiewicz, BarbaraHribal, MartaSzmodis, WhitneyHrvoj-Mihic, BrankaSzőllősi, ÁgnesHsiao, Hao-YuanSzopa, AleksandraHsieh, Shu-ShihSzumlinski, KarenHsieh, Tung-HsunTabarés-Seisdedos, RafaelHu, HuiTabatabaei-Jafari, HosseinHu, RuifengTaber, Katherine JohansenHu, XiaosuTabolacci, ElisabettaHu, ZhengqingTabuchi, MasashiHuang, Chung-GueiTadesse, TeferaHuang, HantaoTaipa, RicardoHuang, Helen J.Takaki, ManabuHuang, LeiTakayama, YutaroHuang, Shar Yin NaomiTakeda, ToshinobuHuang, ZiruiTames, JoseHubáčková, ŠárkaTamura, HidekiHucho, Tim B.Tan, LongzhiHudomiet, PéterTan, Seng CheeHumer, ElkeTanaka, MasaruHundza, SandraTanaka, NaoroHuotari, MattiTang, LingfeiHutson, Thomas H.Tang, ShiyuHuyck, Julia JonesTarantini, StefanoHwa, LaraTarantino, SamuelaHyman, James M.Tarraga-Minguez, RaulHyngstrom, Allison S.Tassé, MarcIacoangeli, AlfredoTateishi, KensukeIacono, DiegoTayeb, ZiedIansek, RobertTaylor, Briana J.Iasevoli, FeliceTaylor, GemmaIbrahim, El ChérifTeichert, TobiasIgolkina, Anna A.Tekok-Kilic, AydaIlag, Leodevico L.Tekriwal, AnandIlchibaeva, TatianaTéllez-Zenteno, José FranciscoIlieva, MirolybaTen Brink, Antonia F.Illiano, PlacidoTender, Gabriel ClaudiuImai, TakaoTerada, SeishiImani, MahdiTeramoto, ShinjiImbach, LukasTercero, Jesús SalidoImperatori, ClaudioTermsarasab, PichetIneichen, ChristianTeruel-Marti, VicentIngre, CarolineTeruyuki, TanakaIngrid, TonhajzerováTessadori, JacopoInvitto, SaraTeti Mayer, JulianaIrwin, ZacharyThaut, Michael H.Ishikawa, YasuyukiTheys, CatherineIsidori, AndreaThiele, CarstenIsoard-Gautheur, SandrineThomas, HallieIsraeli-Korn, Simon DanielThome, JohannesIto, TadashiThyagarajan, DominicIwano, TomohikoTiemeier, HenningIwasaki, MasakiTifft, CynthiaIwata, YukoTijms, Betty M.Iyer, JananiTimmerman, VincentJacobucci, RossTinajero, CarolinaJaeger, AllisonTisserand, RomainJajcay, NikolaToma, MilanJakaria, Md.Tomczyk, ŁukaszJakimovski, DejanTonacci, AlessandroJalnapurkar, Isha R.Tonetti, LorenzoJames, MorganTonhajzerova, IngridJang, HyungseokToribio-Mateas, MiguelJang, WooyoungToro, CamiloJankowska, MilenaTorres, NapoleonJansen, PetraTorres-Filho, Ivo PontesJansson, DeidreTorres-Perez, Jose VicenteJaquess, KyleTorres-Sanchez, SoniaJarvis, ErichToscani, MatteoJasien, JoanTrafalis, Dimitrios T.Jasoliya, MittalTran, YvonneJaworska, NataliaTrapero, IsabelJayanthi, SankarTrask, SydneyJean, WalterTraub, Richard J.Jeanblanc, JeromeTrauner, DorisJean-Quartier, ClaireTremblay, FrançoisJensen, Rigmor HøjlandTrenado, CarlosJeong, JaeseungTriaca, VivianaJeong, Young-SeobTripathi, Jitendra KumarJerzemowska, GrażynaTripi, GabrieleJeter, Cameron B.Troup, LucyJi, YuelongTrujillo, Logan T.Jiang, ChengTsai, Chia-LiangJianu, Dragos CatalinTsai, Rong-KungJillella, DineshTseng, PhilipJimenez-Ferrer, ItziaTsetsos, FotisJin, DiTsivgoulis, GeorgiosJin, HongTünnermann, JanJin, XinTunon, JoseJiralerspong, TrongmunTural, ÜmitJohansson, BirgittaTurati, ChiaraJohnson, AlisaTurri, MaraJohnstone, StuartTvrdik, PetrJollans, LeeTyburski, ErnestJones, CherylTyler, Richard S.Jorge, JoaoTyurin-Kuzmin, PyotrJoshi, CharutaTyynismaa, HennaJuengst, Shannon B.Ubaldi, MassimoJulkunen, PetroUchino, HarutoJumaa, Mouhammad A.Uchmanowicz, IzabellaJung, JinseiUddin, ZakirKafkas, AlexUhl, EberhardKahaki, Seyed Mostafa MousaviUllah, ZahidKaiser, Thomas M.Upadhya, Manoj AtmaramjiKalashnikova, MarinaUpadhyay, NeerajKallioniemi, ElisaUram, ŁukaszKambanaros, MariaUrban, JillianKambara, ToshimuneUttal, DavidKamholz, JohnUwe, MayerKamijo, KeitaVachata, PetrKaminska, MartaVaismoradi, MojtabaKaplan, BatiaVajapeyam, SridharKaramacoska, DianaValensin, DanielaKarhu, JariValentín, AntonioKarim, AhmedVallicelli, Elia ArturoKarimi-Rouzbahani, HamidVan De Ruit, MarkKarlsson, FredrikVan Den Buuse, MaartenKarp, Jonathan D.van der Ham, InekeKataki, MariaVan Der Wardt, VeronikaKatneni, UpendraVan Der Zee, EviKatrinli, SeymaVan Gool, Stefaan W.Katsanos, Aristeidis H.Van Grevenynghe, JulienKatsila, TheodoraVan Oosterwijck, JessicaKatsinelos, TaxiarchisVan Roon, Willeke M. C.Katuchova, JanaVan Schependom, JeroenKauer, Julie A.Vann, Seralynne D.Kaufmann, Walter E.van't Wout, MaschaKaul, MarcusVanutelli, Maria ElideKawala-Sterniuk, AleksandraVarela-Nieto, IsabelKawasaki, HirotoVarinen, AleksiKawasaki, TsubasaVarma, ParulKazufumi, SuzukiVasile, FrancescaKazumata, KenVasileios, KokkinosKeehn, BrandonVater, ChristianKeenan, Julian PaulVaverka, MiroslavKeijzer, MerelVelázquez Coccia, FernandaKelly, Claire M.Veldeman, MichaelKelly, RachelVelev, Dimiter GeorgievKennedy, Donna L.Velikova, TsvetelinaKern, DrewVelisek, LiborKernbach, JuliusVelnar, TomažKershner, JohnVendruscolo, LeandroKerzel, DirkVenturelli, MassimoKerzel, MatthiasVera Rojas, JaimeKeser, ZaferVercillo, TizianaKesler, ShelliVercoutter-Edouart, Anne-SophieKhaksari, KosarVerde, FedericoKhamassi, MehdiVerrecchia, LucaKhan, Ahmad RazaVetrano, Ignazio GaspareKhan, NababVidal, Pia M.Khan, Zeeshan AhmadVignoli, AglaiaKhattar, Nicolas K.Vijayan, MuraliKheradmand, AmirVilariño-Güell, CarlesKhojasteh, JamVilla, Filippo MariaKhokhar, BilalVink, AnnemiekeKhoutorsky, ArkadyVita, GiuseppeKichigina, ValentinaVlaicu, Mihaela Bustuchina Kiiski, Hanni S. M.Voellger, BenjaminKilb, WernerVolicer, LadislavKilian, CarolinVolpe, YaryKim, Jeong-YeonVolpicelli, FlorianaKim, Jun HeeVon Lühmann, AlexanderKim, KaVon Wrede, RandiKim, KiyoungVoroshilova, AnzhelikaKim, SooJoungVucic, SteveKIM, Sung JoonVulchanova, Mila DimitrovaKim, Sung-HeeVulturar, RomanaKimura, TadashiWada, MakotoKindy, Mark S.Waddell, JaylynKireev, Maxim V.Waddington, HannahKirov, RoumenWagner, David H.Kirshner, HowardWahl, Anna-SophiaKitner-Triolo, MelissaWais, Peter E.Kjaerulff, OleWaldis, LeaKlaiman, CherylWalenkamp, AnnemiekKlaus, JanaWalitt, BrianKlaver, PeterWalker, Douglas G.Kleczkowska, PatrycjaWalters, JoelKlein, SamWalz, KatherinaKlichowski, MichałWändell, PerKlietz, MartinWang, Grace Y.Klimek, MarkusWang, JinsungKluczyk, AlicjaWang, JunKnechtle, BeatWang, LiyeKnight, HelenWang, MaosenKnipper, MarliesWang, ShaobinKnotkova, HelenaWang, Tang-ChuanKobeissy, FirasWarnert, Esther A. H.Koda, MasaoWashburn, David A.Kodali, MaheedharWatson, PoppyKoerner, TessWaung, MaggieKoga, HisashiWeber, EricaKohyama, JunWee, Sang OukKoirala, NabinWeerasinghe, GihanKoltz, Michael T.Weglarz, WladyslawKong, JunWeigard, AlexanderKoppen, IlanWeil, Alexander G.Kopyta, IlonaWeinstein, Aviv M.Korb, FranziskaWeissman, David G.Korecky-Kröll, KatharinaWeizman, AviKornblith, EricaWellington, CherylKörner, AnitaWelsh, C. JaneKorres, George S.Werchan, Denise M.Kostaras, PanagiotisWerker, JanetKota, SrinivasWężyk, MichalinaKotlȩga, DariuszWhite, Stormi PulverKotloski, RobertWhitman, BarbaraKotter, Mark R.Wiatrak, BenitaKourtesis, PanagiotisWiderska-Chadaj, Zaneta S.Koutras, AthanasiosWienecke, JacobKoutsis, GeorgiosWiest, Michael C.Kovacs, KristofWilder-Smith, EinarKovacs, RichardWilkinson, Larrell L.Koyuncu, SedaWilliams, Justin H. G.Kozhevnikova, OyunaWilliams, Robert L.Kozlowska, KasiaWilliams, SianKraan, ClaudineWills, TiffanyKraft, FlorianWilson, DuncanKraus, ElisabethWilson, MarcusKrautwurst, DietmarWilson, SueKrol, ManonWirz, LisaKrug, IsabelWiseheart, RebeccaKrukar, JakubWitney, AliceKrupa, MichałWittevrongel, BenjaminKrutki, PiotrWlaź, PiotrKruuse, ChristinaWnuk, AgnieszkaKrzywoszynska, KarolinaWohl, StefanieKrzyzak, Artur TadeuszWojcik, GrzegorzKrzyżewski, RogerWolf, ErikaKu, BonchoWolf, Oliver T.Kuan, Chia-YiWolfer, SaschaKucharska-Newton, AnnaWoll, BencieKujawska, MałgorzataWollesen, BettinaKumamoto, EiichiWolter, TilmanKumar, GauravWong, Lee-Jun C.Kumar, Kishore RajWoodbury-Smith, MarcKumar, RajWoodcock, KateKumar, SuneelWorkman, Craig D.Kumar, VijayWright, David L.Kumar, VinodWu, QihuiKumazaki, HirokazuWu, TianyingKummer, KaiWu, WeiKung, David K.Wysiadecki, GrzegorzKung, KarsonWyver, ShirleyKung, Woon-ManXi, Zheng-XiongKurada, LalithaXiang, HenryKuras, YuliyaXie, HehuangKurihara, MasanoriXie, RuiliKurimoto, TakujiXie, WanzeKurrasch, DeborahXing, ZhuoKutafina, EkaterinaXu, HuichunKuwahara, TomokiXu, KuiKuwaki, TomoyukiXu, WilliamLa Torre, AnnaYabluchanskiy, AndriyLach, GilliardYadav, DhananjayLacroix, AgnèsYagmurlu, KaanLaeng, BrunoYahia-Cherif, LydiaLafrenaye, AudreyYamamoto, TatsuyaLagares, AlfonsoYamasaki, BriannaLahmiri, SalimYamauchi, TakashiLai, YanhaoYang, ChunzhangLalonde, RobertYang, Jenq-LinLalouni, MariaYang, JialeLam, VirginieYang, Jong-ChulLampert, AngelikaYang, LixiaLancaster, Hope SparksYao, HiroshiLand, BenjaminYao, LiLandi, SilviaYates, Nathanael JamesLane, AlisonYavagal, DileepLane, Hsien-YuanYe, MingyuLanza, GiuseppeYe, XinLanzone, JacopoYegla, Brittney A.Larrañaga, PedroYeh, I. JuLarson-Prior, Linda J.Yip, Bon HamLasley, StephenYokoyama, ShigeruLatif, NidaYoneoka, YuichiroLattanzi, SimonaYoneyama, MasanoriLau, Wallis C. Y.Yong, Voon WeeLaukka, Erika JonssonYoong, MichaelLauriello, MariaYoung, KymberlyLautrey, JacquesYoussef, HibaouiLavidor, MichalYoussef, NagyLaw, PhillipYoussofzadeh, VahabLe Merrer, JulieYu, Jane JieLeasure, J. LeighYu, JunweiLeaver, Helen AnneYu, YanboLecciso, FlaviaYuan, XiaobingLechpammer, MirnaYue, John K.Lee, Chia-ChengYufik, Yan M.Lee, Chung-HaoYui, KunioLee, Darrin JasonZaben, MalikLee, HeonsooZafar, AmadLee, Hyeon KyuZagalaz-Anula, NoeliaLee, JisooZaghloul, NahlaLee, Young JuZahr, Natalie M.Léger, Pierre-MajoriqueZainul, ZarinLegg, LynnZając-Lamparska, LudmiłaLeineweber, MatthewZajic, MatthewLemée, Jean-MichelZakrocka, IzabelaLemon, Jennifer A.Zamani, AkramLempiäinen, JuhaZammit, AndreaLench, Daniel H.Zaniboni, AlbertoLeone, CaterinaZanker, JohannesLeppert, WojciechZapico, Sara CasadoLepping, Rebecca J.Zaretsky, ElenaLeroi, IracemaZarnowska, Iwona MariaLesca, GaëtanZatelli, Maria ChiaraLethaus, BerndZebrowski, Jan J.Leung, Doris Y. P.Zedde, Maria LuisaLevante, AnnalisaZefferino, RobertoLevitin, Daniel J.Zeier, ZaneLevone, Brunno Rocha Zeighami, YasharLevshina, NataliaZennifa, FadillaLewis, ChandaniZerhouni, OulmannLi, HuiquanZhang, FanglueLi, Ka WanZhang, HaoSuLi, SunanZhang, JinshengLi, YikeZhang, JixiangLiang, JunZhang, MengliangLiang, Nu-ChuZhang, YangLiang, TiebingZhang, YuLibertus, KlausZhao, MinLichtenthaler, StefanZhao, MingjunLietzau, GrazynaZhao, QingnanLihui, WangZhigalov, AlexanderLiker, Mark A.Zhou, JunhongLim, JiseunZhou, XiaohaiLin, Ai-LingZhou, YanLin, Che-WeiZiegler, DanaLin, HongZille, MariettaLin, ZhichengZito, Giuseppe AngeloLind, GöranZivotofsky, AriLindroth, HeidiZschorlich, Volker RudolfLintas, AlessandraZuber, SaschaLipp, IlonaZucconi, MarcoLippens, GuyZup, SusanLippert, Rachel N.Żygierewicz, JarosławLisi, Antonella


